# Closing the loop: a needs-assessment informed model for embedding a non-allergist penicillin allergy de-labelling service in acute medicine, a quality improvement report

**DOI:** 10.1093/jacamr/dlag244

**Published:** 2026-11-05

**Authors:** Philip Toner, Annabel Glass, Cathal Donnelly, Katherine Garnett, Mark Mchaffie, Sean Quinn, Christopher Hillen, Jonathan Mullan, Yazan H Mustafa, Connor Maguire, Sean Faulkner, Bernice Ng, Aaron Brady

**Affiliations:** Acute Medical Unit, Royal Victoria Hospital, Belfast Health and Social Care Trust, Grosvenor Road, Belfast, Northern Ireland, UK; Centre for Medical Education, School of Medicine, Dentistry and Biomedical Sciences, Queen's University Belfast, 97 Lisburn Road, Belfast, Northern Ireland, UK; Pharmacy Department, Belfast Health and Social Care Trust, Grosvenor Road, Belfast, Northern Ireland, UK; Acute Medical Unit, Royal Victoria Hospital, Belfast Health and Social Care Trust, Grosvenor Road, Belfast, Northern Ireland, UK; Acute Medical Unit, Royal Victoria Hospital, Belfast Health and Social Care Trust, Grosvenor Road, Belfast, Northern Ireland, UK; Acute Medical Unit, Royal Victoria Hospital, Belfast Health and Social Care Trust, Grosvenor Road, Belfast, Northern Ireland, UK; Acute Medical Unit, Royal Victoria Hospital, Belfast Health and Social Care Trust, Grosvenor Road, Belfast, Northern Ireland, UK; Acute Medical Unit, Royal Victoria Hospital, Belfast Health and Social Care Trust, Grosvenor Road, Belfast, Northern Ireland, UK; Acute Medical Unit, Royal Victoria Hospital, Belfast Health and Social Care Trust, Grosvenor Road, Belfast, Northern Ireland, UK; Acute Medical Unit, Royal Victoria Hospital, Belfast Health and Social Care Trust, Grosvenor Road, Belfast, Northern Ireland, UK; Acute Medical Unit, Royal Victoria Hospital, Belfast Health and Social Care Trust, Grosvenor Road, Belfast, Northern Ireland, UK; Centre for Medical Education, School of Medicine, Dentistry and Biomedical Sciences, Queen's University Belfast, 97 Lisburn Road, Belfast, Northern Ireland, UK; Acute Medical Unit, Royal Victoria Hospital, Belfast Health and Social Care Trust, Grosvenor Road, Belfast, Northern Ireland, UK; Acute Medical Unit, Royal Victoria Hospital, Belfast Health and Social Care Trust, Grosvenor Road, Belfast, Northern Ireland, UK; Pharmacy Department, Belfast Health and Social Care Trust, Grosvenor Road, Belfast, Northern Ireland, UK; School of Pharmacy, Queen's University Belfast, 97 Lisburn Road, Belfast, Northern Ireland, UK

## Abstract

**Objectives:**

To describe and evaluate a needs-assessment informed model for embedding a non-allergist penicillin allergy de-labelling (PADL) service within an Acute Medical Unit (AMU), including stakeholder experience.

**Methods:**

A four-phase quality improvement project in one UK AMU, reported per SQUIRE 2.0. Phase 1: needs-assessment survey of 124 staff (pharmacists, doctors, nurses, immunology staff). Phase 2: a multidisciplinary team translated Phase 1 barriers into a PEN-FAST-guided direct oral amoxicillin challenge (DOC) protocol delivered by acute medicine consultants and pharmacists; design decisions were retrospectively mapped to the capability, opportunity and motivation model for behavioural change and Behaviour Change Wheel. Phase 3: DOC in 60 adult inpatients penicillin allergy labels. Phase 4: post-implementation survey of 54 patients and staff.

**Results:**

Before implementation, 90% of staff anticipated a service would be useful, but identified time, education, safety concerns and staffing as the main barriers. Fifty-nine of 60 patients (98.3%) were de-labelled, with one mild Type 4 reaction but no anaphylaxis. All 21 (35.6%) who needed antibiotics within six months received a penicillin without reported reaction. All 54 post-implementation respondents rated the service extremely useful and would recommend or refer into it; as Phase 1 and Phase 4 cohorts were non-matched, this comparison is indicative only. Patients described years of unwarranted anxiety about antibiotic use that resolved after de-labelling.

**Conclusions:**

A needs-assessment-informed model translated staff-identified barriers into design decisions and was associated with high stakeholder endorsement, durable penicillin use and an under-recognized psychological benefit for patients. It offers a practical, replicable template for non-allergist PADL in acute medicine.

## Introduction

Penicillin allergy labels are estimated to affect approximately 3 million people in the UK, yet more than 90% do not correspond to a genuine IgE-mediated or other clinically significant hypersensitivity reaction.^1–3^ This mislabelling carries substantial consequences for patients and society. For patients, incorrect penicillin allergy labels are associated with prolonged hospital admissions,^4^ increased use of broader-spectrum, less efficacious non-penicillin antibiotics^5–7^ and an increased risk of infection with multidrug-resistant organisms and *Clostridioides difficile*.^4,8^ For society, there is increased healthcare expenditure,^9^ accelerated emergence of antimicrobial resistance (AMR)^3,8^ and a measurable environmental burden.^10^

This problem is highly amenable to correction. A 2026 systematic review of 42 studies (6269 patients) found an average successful PADL rate of 80%, ranging from 67% for direct de-labelling alone to 87% for formal skin testing, with direct oral challenge (DOC) achieving 77%.^11^

This evidence base has been matched by growing political and professional commitment. The EU One Health Action Plan against AMR, and its 2023 Council Recommendation, call for stronger antimicrobial stewardship (AMS).^12^ The European Academy of Allergy and Clinical Immunology’s 2020 position paper offers risk-stratified diagnostic algorithms for beta-lactam hypersensitivity.^13^ In the UK, the government’s Confronting Antimicrobial Resistance 2024–29 national action plan states that access to de-labelling services is essential;^14^ the British Society for Allergy and Clinical Immunology’s (BSACI) 2022 guideline sets out how non-allergist clinicians can deliver PADL,^15^ and National Institute for Health and Care Excellence’s (NICE) CG183 drug allergy guidance provides further support.^16^ Locally, the Northern Ireland Executive Programme for Government commits to tackling AMR.^17^

Despite this endorsement, it remains difficult to establish and sustain a productive de-labelling service.^18,19^ Non-allergist clinicians (doctors, pharmacists and nurses without specialist allergy training) report low knowledge of, and confidence in, risk-stratifying allergy tools, limited training and fear of inducing an adverse reaction, while prescribing an alternative antibiotic remains a simple opt-out.^18,20–22^ Patients may fear the safety of testing, distrust a negative result or continue avoiding penicillin even after successful de-labelling.^23–25^ Even where services launch successfully, sustaining them beyond an initial pilot is a further challenge, and system-level evidence on scalable, durable service design remains sparse.^19,26^ These clinical, patient and organizational barriers may explain why PADL has been slow to translate into embedded routine services.^11,21^

Non-allergist PADL delivered through a stand-alone inpatient pathway has recently been investigated by Powell and colleagues, spanning staff-reported barriers and enablers^22^ and patient experience.^25,27^ By contrast, the Penicillin Allergy Novel De-Labelling in the Acute Medical Unit (AMU) setting (PANDA) service is embedded directly within an AMU, addressing these barriers within routine acute care.^10^ Our study is complementary and indeed addresses a novel approach. We report a single, continuous four-phase cycle from needs assessment to stakeholder evaluation, and formally map staff-identified barriers onto the capability, opportunity and motivation model for behavioural change (COM-B) and Behaviour Change Wheel (BCW) as a reusable design template.

Full clinical, safety, economic and environmental outcomes of the PANDA pilot are reported in a companion publication.^10^ This report focuses on the needs-assessment and design process (Phases 1–2) and stakeholder reception (Phase 4), summarizing Phase 3 only as context; this aspect of implementation is rarely documented yet directly relevant to units seeking to establish similar services.

## Aim

To describe and evaluate the development of a non-allergist, AMU-embedded PADL service across four sequential phases: (i) needs assessment; (ii) service design; (iii) implementation and (iv) stakeholder evaluation.

## Patients and methods

This report is structured according to the Standards for Quality Improvement Reporting Excellence (SQUIRE 2.0) guidelines.^28^ The project was undertaken in the AMU of the Royal Victoria Hospital, Belfast Health and Social Care Trust (BHSCT), across four sequential phases (Figure 1).

**Figure 1.**
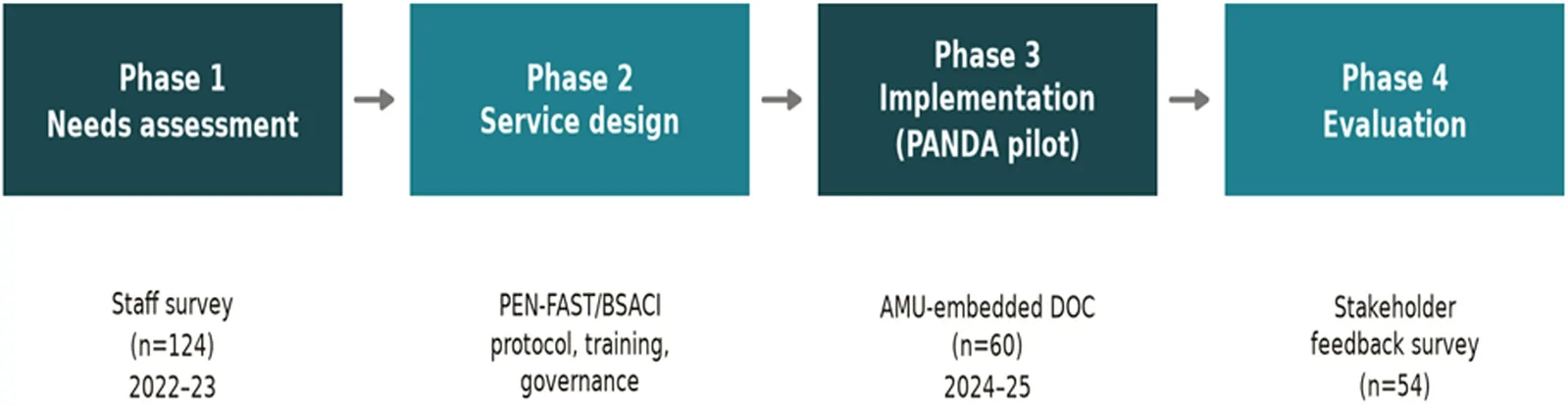
Four-phase service development timeline.

### Phase 1—needs assessment

An electronic cross-sectional questionnaire (17 items: 3 open-ended, 10 closed, 4 Likert-scale) was distributed via Microsoft Forms to 320 clinical pharmacists, medical and surgical doctors, nurses and immunology clinical staff across BHSCT, using departmental email distribution lists with one reminder. The questionnaire assessed: (i) awareness and understanding of the term PADL; (ii) views on the prevalence and consequences of inaccurate penicillin allergy labels; (iii) ability to distinguish a true allergy from an intolerance or other adverse effect; (iv) prior clinical experience of penicillin-related anaphylaxis; and (v) anticipated usefulness of, and likelihood of referring into, a new PADL service. It was piloted with a pharmacist, a nurse and a doctor before distribution, and was voluntary and anonymous. Responses were analysed descriptively and free text thematically (see Analysis).

### Phase 2—service design

Findings from Phase 1 directly informed service design (Table 1), led by a multidisciplinary group of acute medicine consultants, clinical pharmacists, AMU nursing staff and the regional immunology team. A risk-stratified DOC protocol (Figure 2) was developed in accordance with the BSACI guideline for non-allergists,^15^ combined with the PEN-FAST clinical decision rule.^29^ PEN-FAST scores how recent the reaction (≤5 years), anaphylaxis/angiooedema or severe cutaneous adverse reaction and need for treatment. A score <3 identifies low-risk patients, with a negative predictive value of 96.3% in its validation cohorts.^29^

**Figure 2.**
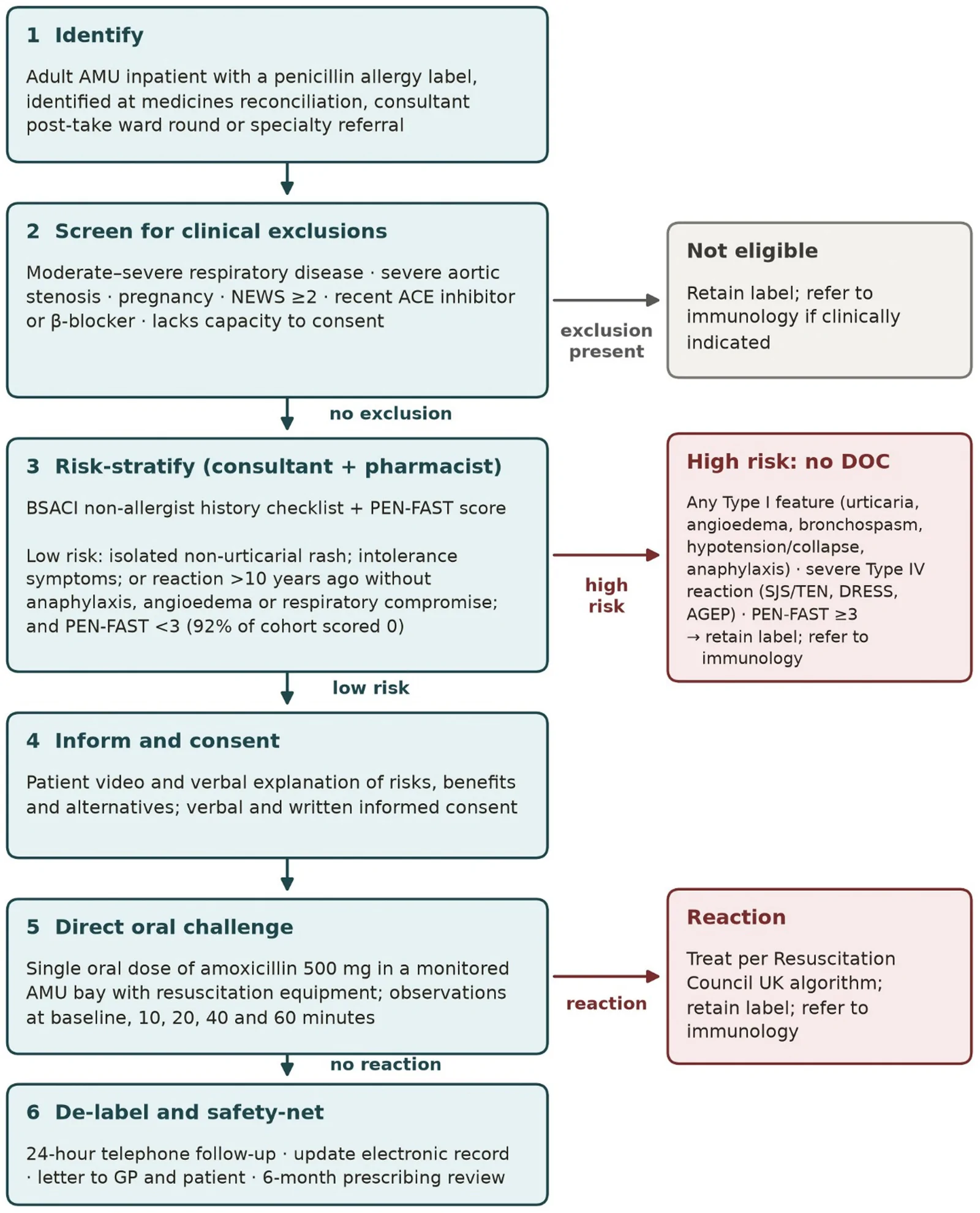
PANDA risk satisfaction and DOC protocol. SJS/TEN, Stevens–Johnson syndrome/toxic epidermal necrolysis.

**Table 1.** Mapping of the top pre-implementation barriers identified in Phase 1 to the corresponding design decision adopted in Phases 2–3

Pre-implementation barrier (Phase 1, n = 124)	COM-B domain	BCW function	Phase 2–3 design response
Time constraints (44%)	Opportunity (physical)	Environmental restructuring	Embedded within existing AMU workflow rather than a stand-alone clinic
Education/awareness gaps (31%)	Capability (psychological)	Education, training	Targeted educational videos, simulation training, structured use of PEN-FAST
Patient safety concerns (28%)	Motivation (reflective) and capability	Education, environmental restructuring	Strict exclusion criteria, monitored observation, patient education
Staffing/capacity (26%)	Opportunity (physical/social)	Environmental restructuring	Delivered by existing staff; no dedicated additional personnel
Referral system complexity (16%)	Opportunity (physical/social)	Environmental restructuring	Simplified referral from any speciality, at any admission stage

PEN-FAST was chosen for three reasons. First, it has been prospectively derived and externally validated in European and North American cohorts.^30,31^ Second, in the multinational PALACE randomized trial, DOC in patients scoring <3 was non-inferior to skin testing followed by oral challenge.^32^ Third, the simple matrix takes minutes for non-allergist doctors and pharmacists to complete, without specialist equipment, addressing the Phase 1 barriers of time and knowledge. Finally, it produces a documented, auditable score. Alternatives, such as the BSACI eligibility checklist^15^ and programme-specific risk criteria,^33^ use more complex history-based categories without a validated score; we therefore used PEN-FAST alongside, rather than instead of, the BSACI checklist.

Low-risk histories eligible for DOC were a PEN-FAST score <3 together with an isolated non-urticarial rash, symptoms of intolerance, or a reaction more than 10 years earlier without features of anaphylaxis, angio-oedema or respiratory compromise.^15^ Patients were excluded if they reported any feature suggestive of a Type 1 reaction (including urticaria, angio-oedema, bronchospasm or wheeze, hypotension or collapse, anaphylaxis or a reaction requiring adrenaline or hospital treatment), a severe Type 4 reaction [e.g. Stevens–Johnson syndrome/toxic epidermal necrolysis or drug reaction with eosinophilia and systemic symptom (DRESS)]; moderate-to-severe respiratory disease; severe aortic stenosis; pregnancy; National Early Warning Score (NEWS) ≥2; recent ACE inhibitor or beta-blocker use or inability to give informed consent.^10,15^ Because published low- and high-risk definitions differ, Table 2 compares these criteria with the BSACI guideline and PEN-FAST/PALACE thresholds.^15,29,32^

**Table 2.** Low- and high-risk criteria for direct oral penicillin challenge: BSACI guideline, PALACE and the PANDA protocol

Criterion	BSACI guideline for non-allergists (2022)^15^	PALACE trial^32^	PANDA protocol (this project)^10^
Approach	History-based checklist (eligible histories and exclusions) no numerical score	PEN-FAST score	BSACI-aligned history checklist combined with PEN-FAST score, assessed jointly by an acute medicine consultant and clinical pharmacist
Low-risk/eligible histories	Benign rash (non-urticarial, non-itchy, non-blistering, <24 h, no treatment) >1 h after first dose; childhood rash with no other history; GI symptoms, thrush or other non-allergic symptoms; family history only; penicillin since tolerated; reaction not remembered but reported as not serious	Score <3 (low risk; negative predictive value 96.3%); PALACE enrolled adults scoring <3 for DOC	Isolated non-urticarial rash; intolerance symptoms or reaction >10 years earlier without anaphylaxis, angio-oedema or respiratory compromise and PEN-FAST <3 (92% of cohort scored 0)
Timing of index reaction	Rash within 1 h of first dose excludes; no minimum time since reaction	Reaction ≤5 years ago scores 2 points	Non-benign reactions eligible only if >10 years earlier (stricter than PEN-FAST)
Immediate (Type I) features	Any feature excludes swelling, urticaria, angio-oedema, shortness of breath, wheeze, loss of consciousness, collapse; reaction needing hospital treatment or adrenaline	Anaphylaxis/angio-oedema scores 2; urticaria is not scored separately	Excluded: any feature suggestive of a Type I reaction, including urticaria, angio-oedema, bronchospasm/wheeze, hypotension or collapse, anaphylaxis or reaction needing adrenaline/hospital treatment
Severe delayed (Type IV) reactions	Excluded: blistering, skin peeling, mucosal involvement, purpura, rash >24 h or >10% body surface area	Severe cutaneous adverse reaction scores 2	Excluded: severe Type IV reactions (e.g. SJS/TEN, DRESS)
Clinical exclusions	Severe or uncontrolled asthma; severe COPD; severe aortic stenosis; acute instability (respiratory or cardiac failure, arrhythmia); pregnancy; unable to consent	Not part of the score	Moderate-to-severe respiratory disease; severe aortic stenosis; pregnancy; NEWS ≥2; unable to consent
Medication exclusions	Not listed in the eligibility checklist	Not part of the score	Recent ACE inhibitor or beta-blocker
Challenge and follow-up	Single- or divided-dose challenge with index penicillin (amoxicillin if unknown); prolonged (3-day) challenge if index reaction followed later doses or timing unknown; BP, HR and SpO_2_ before and after each dose	PALACE: direct oral penicillin challenge; primary outcome within 1 h; immune-mediated events recorded for 5 days	Single oral amoxicillin 500 mg; observations at baseline, 10, 20, 40 and 60 min; 24-h telephone follow-up; 6-month prescribing review

BP, blood pressure; COPD, chronic obstructive pulmonary disease; GI, gastrointestinal; HR, heart rate; SJS/TEN, Stevens–Johnson syndrome/toxic epidermal necrolysis; SpO_2_, oxygen saturation.

Governance approval followed BHSCT Drugs and Therapeutics Committee assent. Before launch, all PANDA staff (acute medicine consultants, clinical pharmacists and AMU nurses) completed training comprising: (i) a face-to-face session on penicillin allergy phenotypes, the BSACI checklist and PEN-FAST scoring using worked case histories and (ii) in-situ, scenario-based simulation in the AMU, using a simulated patient. Rehearsing recognition and escalation of reactions to a DOC, from isolated urticaria or angio-oedema, through bronchospasm, to anaphylaxis with hypotension, and delivery of the Resuscitation Council UK anaphylaxis algorithm.^34^ Recognition and safety-netting of delayed (Type IV) reactions were also covered.

Short (3-min) educational videos, piloted with clinical staff and medical students, were designed to address the issues and concerns identified in Phase 1, in two separate sets. Staff videos, for AMU doctors, nurses and pharmacists, covered identification and treatment of allergic reactions, the reasons for and impact of false allergy labels, the PEN-FAST rationale, PANDA procedures and safety-netting. Patient videos, for patients being offered DOC and viewed before consent, explained why antibiotics differ, the impact of a false allergy label, the importance of AMR, what the PANDA procedure involves and how to recognize and report an allergic reaction.

Coding was applied retrospectively to characterize the mechanisms underlying design decisions. Two authors independently coded each Phase 1 barrier and its corresponding Phases 2–3 design response against the COM-B components and BCW intervention functions, using a coding frame derived from Michie et al.^35^ Codes were compared at a consensus meeting, and discrepancies were resolved by discussion with reference to the source survey data.

### Phase 3—implementation (the Panda pilot)

Recruitment was opportunistic, on weekdays during staffed hours, and out-of-hours admissions were reviewed the next working day. Suitable adult AMU inpatients were identified during routine medicines reconciliation, consultant post-take ward rounds or specialty referrals. Then risk assessed jointly by an acute medicine consultant and a clinical pharmacist. A single 500-mg oral dose of amoxicillin was prescribed by the responsible doctor or pharmacist and administered by AMU nursing staff in a monitored bay with immediate access to resuscitation equipment. Physiological observations were recorded at baseline and at 10, 20, 40 and 60 min, followed by a structured 24-h telephone follow-up for delayed reactions.^10^ Outcomes were communicated formally to the patient and their general practitioner and recorded in the electronic record. Subsequent antibiotic prescribing was reviewed for six months after de-labelling using the regional electronic care record.^10^

Patients were approached only if they had capacity to consent. The assessing consultant or pharmacist explained the purpose of de-labelling, the procedure, the small risk of an immediate or delayed reaction (including anaphylaxis), the safeguards in place and the alternatives (continued penicillin avoidance or referral to the immunology service). Patients had the opportunity to ask questions and could decline without any effect on their care. Verbal and written informed consent was obtained before DOC, including consent for use of anonymized follow-up.

### Phase 4—post-implementation stakeholder evaluation

An anonymous structured feedback survey (8 items, mixed Likert-scale and free text) was distributed to the 156 stakeholders who had encountered the PADL service, including patients/service users, doctors, nurses and pharmacists and patients via email. Questions mirrored Phase 1 and covered the role/context of contact; perceived usefulness; likelihood of recommending or referring into the service; confidence recognizing symptoms of an allergic reaction; perceived significance of inaccurate penicillin allergy labelling for patients and the National Health Service; effectiveness of specific service elements; and whether the service met expectations. Free-text responses were analysed thematically using Braun and Clarke’s six-phase approach.^35–37^ Two authors independently coded responses and then agreed themes by discussion, with a third author available to resolve disagreements.

### Outcome measures

Phase 1: baseline awareness, attitudes and anticipated barriers/enablers among staff.

Phase 4: stakeholder-rated usefulness, likelihood to recommend/refer, confidence, perceived problem significance, effectiveness of service components and expectation-matching.

Cross-phase: comparison of related attitudinal constructs before and after implementation, reported as exploratory only.

Feasibility, safety, subsequent antibiotic prescribing patterns with exploratory economic and environmental impact are reported in the companion paper and referenced here only as contextual, supportive data.

### Analysis

Phase 1 and Phase 4 survey data were analysed descriptively (proportions per response category, with 95% Wilson CIs). Because Phase 1 (staff, *n* = 124) and Phase 4 (staff and patients, *n* = 54) surveyed different, non-matched cohorts using related but not identical wording, before/after comparisons (Figure 4) are not a formally powered controlled comparison. Fisher’s exact tests are reported only as an exploratory, hypothesis-generating check and should not be read as confirmatory.

### Ethics and governance

The Phase 1 needs assessment received ethical approval from the Ulster University Biomedical Sciences Ethics Filter Committee (a co-author’s academic affiliation). The Phase 3 clinical service was conducted as a service evaluation and development initiative and approved under BHSCT governance processes; per UK Health Research Authority guidance, it did not require formal research ethics review. All patients undergoing DOC provided informed consent as described above. The Phase 4 survey was conducted as anonymous, voluntary service evaluation in accordance with BHSCT clinical audit/Quality Improvement governance.

## Results

### Phase 1: needs assessment (*n* = 124)

There were 124 responses (124/320 emailed; response rate 39%): pharmacists (50.8%), nurses (27.4%), doctors (16.1%) and immunology staff (5.6%). Awareness of the term ‘penicillin allergy de-labelling’ (PADL) was 50% overall, lowest among nurses (20.6%) and doctors (45%), and highest among immunology staff (85.7%) and pharmacists (63.5%). Among those aware, 98% could correctly define it. Awareness had mainly been acquired via colleagues (46%) or self-learning (33%), with only 8% citing university teaching.

Most respondents [91%, 95% confidence interval (CI) 84.8%–95.0%] considered inaccurate labelling an important issue, and 68% doubted that all patients reporting a penicillin allergy had a true allergy. However, only 46% could correctly identify all features distinguishing a true allergy from a side effect, and 71.8% had never personally managed penicillin-related anaphylaxis.

Support for a new service was strong: 90% anticipated it would be useful (95% CI 83.8%–94.4%) and 85.5% said they would likely refer (95% CI 78.2%–90.6%). Respondents favoured referral from all professional groups, at any stage of admission, with a preference for referral on admission. The most frequently cited barriers were time (44%), education/awareness (31%), patient safety concerns (28%), staffing/capacity (26%) and referral complexity (16%) (Figure 3; Table 1).

**Figure 3.**
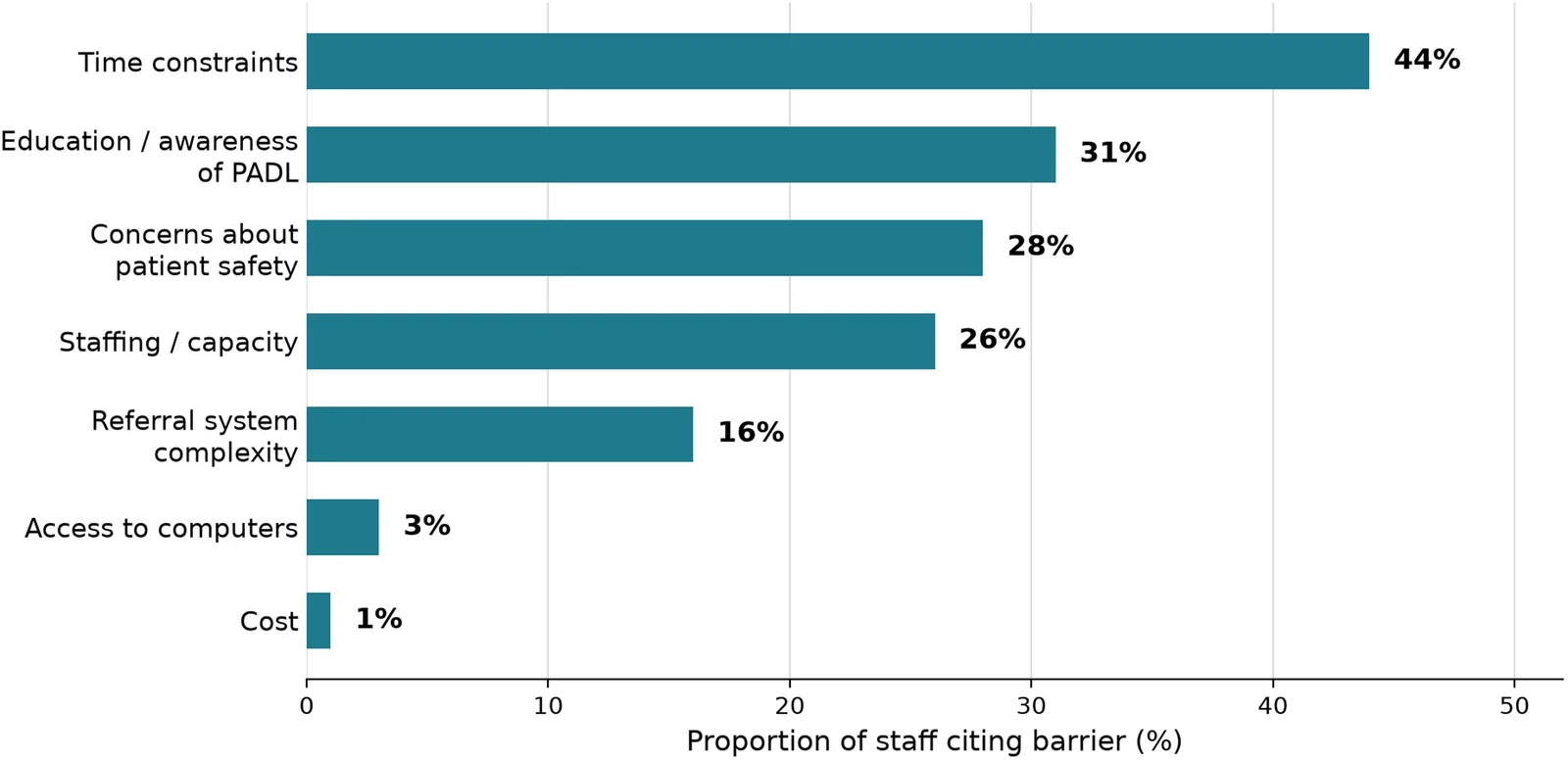
Barriers to new PADL service identification pre-implementation (staff survey, *n*=124).

### Phases 2–3: service design and implementation

Table 1 maps each of the top pre-implementation barriers identified in Phase 1 to the corresponding design decision adopted in Phases 2–3.

Recruitment was guided by strict risk stratification, a deliberate response to the patient safety concerns identified in Phase 1. Of 62 patients approached, 60 (mean age 56, range 27–83) underwent DOC (one refused consent, one opted to postpone). Ninety-two percent scored PEN-FAST 0 (very low risk). Fifty-nine (98.3%, 95% CI 91.1%–99.7%) were successfully de-labelled, with one mild delayed Type IV hypersensitivity reaction but no anaphylaxis. De-labelling was durable; of the 59 de-labelled patients, 21 (35.6%) required antibiotics during the six-month follow-up, and all received a penicillin-based agent without reported adverse reaction.^10^

This de-labelling rate lies within the upper end of the 32%–99% range reported in the 42-study systematic review^11^ (Table 3) and is best framed as consistent with, rather than exceeding, the published evidence, given the small sample and strict risk selection. Full effectiveness, safety and cost analyses appear in the companion PANDA publication.^10^

**Table 3.** Comparative effectiveness of PADL strategies: published evidence versus this project

Strategy	Effectiveness	95% CI/range	Source
Direct de-labelling alone (risk stratification only)	67%	—	Nurnberg et al.^11^
DOC, pooled	77%	—	Nurnberg et al.^11^
Direct de-labelling + oral challenge	80%	—	Nurnberg et al.^11^
Skin testing + oral challenge	84%	—	Nurnberg et al.^11^
Skin testing alone	87%	—	Nurnberg et al.^11^
All strategies pooled (42 studies, *n* = 6269)	80%	32%–99% (range)	Nurnberg et al.^11^
**AMU-embedded PEN-FAST-guided DOC (this project, *n*** **=** **60)**	**98.3%**	**91.1%–99.7%**	**PANDA** ^10^

### Phase 4: post-implementation stakeholder evaluation (*n* = 54)

There were 54 responses (54/156 emailed; response rate 35%): patient/service users (37%), doctors (43%), nurses (7%), pharmacists (9%) and other healthcare professionals (4%). The 156 stakeholders targeted had direct experience of the service as users, AMU staff or referrers.

All 54 respondents (100%, 95% CI 93.4%–100%) rated the service extremely useful and were very likely to recommend or refer into it. Ninety-eight percent (53/54, 95% CI 90.2%–99.7%) rated inaccurate labels a very significant problem for patients and the NHS, and 96% rated accurate allergy status as having a very significant effect on healthcare. Confidence recognizing allergic-reaction symptoms were high (59% extremely confident, 35% somewhat confident and 6% neutral). All four assessed service components (location, consent, referral pathway, staff) were rated somewhat or extremely effective by most respondents. Ninety-three percent (50/54) reported the service completely met expectations, a further 6% mostly; none reported it fell short.

In free-text themes the staff, particularly doctors, identified structural sustainability barriers (referral-pathway formalization, funding) that patients did not raise. Eleven doctors (48%) commented on the clinical/AMS value of the service, 7 (30%) suggested expanding capacity, 6 (26%) called for a formal electronic referral pathway and 4 (17%) wanted the service made permanent and funded. Pharmacists (*n* = 5) gave similar responses. All four nurses reported satisfaction with no suggested improvements.

Patient themes centred on satisfaction: 12 patients (60%) expressed complete satisfaction, 6 (30%) praised staff and care quality and 5 (25%) called for wider availability. A further theme was the impact of the label on patients’ daily life and mental health, corroborating previous reports.^25^ Eight patients (40%) reported frustration at having carried an inaccurate label unnecessarily for decades. Others highlighted underlying anxiety: ‘a lifetime of being terrified of being given penicillin was all in vain’; ‘constantly being worried the doctors don’t know my allergy status is now over’; ‘I’ve spent weeks in hospital due to this allergy status’; ‘everything is more complex with an allergy’.

### Before-and-after comparison

Figure 4 summarizes three attitudinal items assessed in both phases, with 95% Wilson CIs and exploratory Fisher’s exact *P* values. Reported usefulness rose from 90.3% (95% CI 83.8%–94.4%) to 100% (95% CI 93.4%–100%; *P* = 0.019), and likelihood of engagement from 85.5% (95% CI 78.2%–90.6%) to 100% (95% CI 93.4%–100%; *P* = 0.0019). Perceived problem significance rose from 91.1% (95% CI 84.8%–95.0%) to 98.1% (95% CI 90.2%–99.7%; *P* = 0.11). As the cohorts were non-matched and differed in composition, these figures are a descriptive, hypothesis-generating signal only. The uniformly maximal Phase 4 ratings are also consistent with non-response bias favouring satisfied stakeholders.

**Figure 4.**
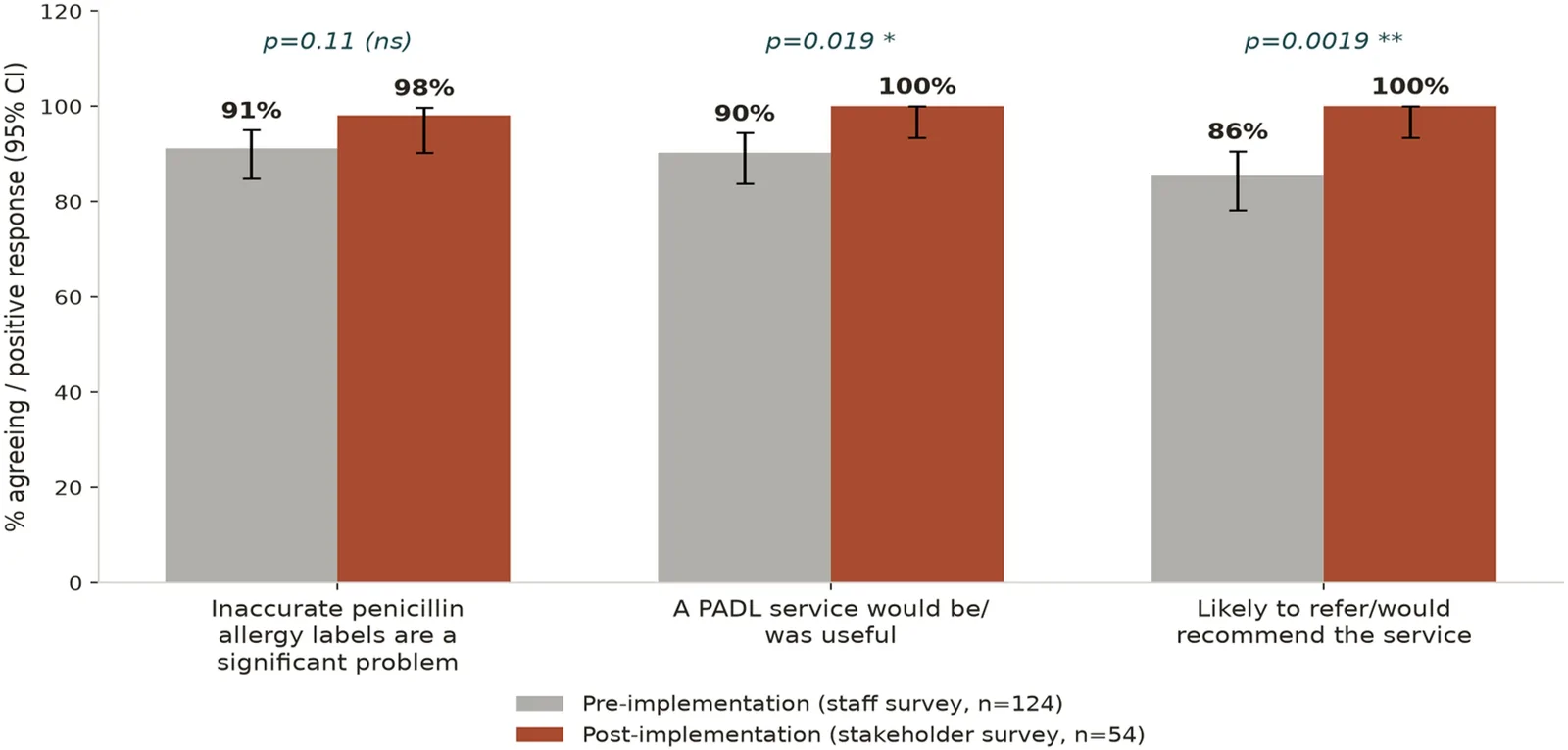
Stakeholder perception before and after service implementation.

## Discussion

This project describes the full development cycle of a non-allergist, AMU-embedded PADL service, from baseline needs assessment through design, implementation and structured stakeholder evaluation.

### Needs assessment to design

The project’s main contribution is showing how a structured baseline needs assessment translated into specific, testable design decisions (Table 1). Time, staffing and referral-complexity concerns were addressed by embedding PADL within existing AMU workflow and ward-round activity, using existing staff, rather than a stand-alone clinic. Education and allergy-history gaps were addressed through simulation training, targeted videos and structured PEN-FAST use, and safety concerns through strict exclusion criteria, monitored observation and resuscitation access. Characterized against COM-B/BCW, most design adjustments altered opportunity (environmental restructuring) rather than capability or motivation, suggesting that sustainability may stem largely from removing structural barriers, consistent with previous reviews.^19,21^ This complements process-evaluation work from a stand-alone inpatient pathway,^22,27^ our contribution is the formal bridge from specific staff-identified barriers to specific design decisions,^36^ offered as a generalizable template.

### Hidden psychological impact of allergy status

This project revealed a dimension of mislabelling rarely captured in effectiveness studies, namely a silent psychological toll on patients living with an inaccurate label. Service users described years of unwarranted fear around antibiotic administration, anxiety during unrelated admissions and a sense that ‘everything is more complex’. That a single, low-risk intervention could reframe ‘a lifetime of being terrified’ as ‘all in vain’ suggests the harms of mislabelling extend beyond antibiotic choice and AMR metrics. This corroborates qualitative findings from a non-allergist inpatient pathway in another UK centre^25^ and extends them to an AMU-embedded context, strengthening the case for framing de-labelling as a low-cost improvement to patient wellbeing as well as an AMS intervention.

### Stakeholder reception in context

Pre-implementation staff support was already favourable but bounded (90.3% anticipated usefulness; 85.5% anticipated referral). Once the service was running, both figures reached 100% among Phase 4 respondents, alongside a smaller, non-significant rise in perceived problem significance (91.1%–98.1%). We interpret this cautiously as an indicative, hypothesis-generating signal rather than a demonstrated effect of implementation on attitudes. A prospective, matched-cohort design is needed to test it.

### Effectiveness and stewardship implications in context

The 98.3% de-labelling rate is consistent with the upper end of the range reported in the systematic review,^11^ but should be interpreted cautiously given the small pilot and strict low-risk selection (92% scoring PEN-FAST 0). This more likely reflects Phase 2 design choices than intrinsic superiority of AMU-embedded DOC. Importantly, de-labelling translated into practice as every patient who subsequently needed antibiotics received a penicillin, which is reassuring given reports that patients and clinicians may continue penicillin avoidance after a negative challenge.^23,24^ The companion publication reports the wider stewardship yield^10^; the process model described here made that achievable within routine AMU workflow, without additional dedicated staffing.

## Implications and future directions

Recently Her Magesty's Coroner Prevention of Future Deaths reports have highlighted the urgent need to roll out PADL to prevent patient harm.^38^ This needs-assessment-to-design model offers a replicable template for other AMUs establishing non-allergist PADL. Future work should prioritize multicentre implementation with prospectively matched pre/post cohorts, alongside prospective health-economic and carbon-footprint evaluation. Respondents also called for integration with electronic patient record systems to support sustainability and spread.

### Limitations

This was a single-centre project. Response rates (39% and 35%) were modest and subject to self-selection bias, so uniformly maximal Phase 4 ratings do not indicate unanimous support. Phase 1 and Phase 4 surveyed different, non-matched cohorts with related but not identical items, so reported *P* values are exploratory only. The de-labelling rate reflects a small, strictly low-risk cohort, not comparable with heterogeneous review populations. Follow-up of subsequent penicillin use was limited to six months and to prescribing visible in the regional electronic record. Finally, the COM-B/BCW mapping was applied retrospectively and may not capture all mechanisms that influenced design decisions.

### Conclusion

A structured, four-phase approach to developing a non-allergist, AMU-embedded PADL service, in which a baseline staff needs assessment directly informed service design, was associated with favourable stakeholder sentiment, durable penicillin use after de-labelling and corroboration of an under-recognized psychological burden of mislabelling. Reporting the full development cycle, rather than clinical outcomes alone, offers a practical, replicable model for embedding PADL as routine acute medicine practice and responds directly to the implementation evidence gap identified in the systematic review literature.

## Data Availability

Anonymized aggregate survey data supporting the findings of this study are available from the corresponding author upon reasonable request, subject to BHSCT information governance approval. Individual-level free-text responses are not available for sharing to protect respondent confidentiality.

## Patient and public involvement

Patients who underwent de-labelling provided informed consent for the procedure and for use of anonymized data in aggregate reporting. Patient/service-user feedback (Phase 4) directly informs the recommendations in this report.
